# Crosstalk between Depression and Dementia with Resting-State fMRI Studies and Its Relationship with Cognitive Functioning

**DOI:** 10.3390/biomedicines9010082

**Published:** 2021-01-16

**Authors:** Junhyung Kim, Yong-Ku Kim

**Affiliations:** 1 Department of Psychiatry, Korea University Guro Hospital, Korea University College of Medicine, Seoul 08308, Korea; jhcabilover@gmail.com; 2 Department of Psychiatry, Yonsei University College of Medicine, Seoul 03080, Korea; 3 Department of Psychiatry, Korea University Ansan Hospital, Korea University College of Medicine, Ansan 15355, Korea

**Keywords:** depression, late-life depression, dementia, Alzheimer’s disease, neuroimaging, resting-state functional magnetic resonance imaging, default mode network, executive control network, salience network

## Abstract

Alzheimer’s disease (AD) is the most common type of dementia, and depression is a risk factor for developing AD. Epidemiological studies provide a clinical correlation between late-life depression (LLD) and AD. Depression patients generally remit with no residual symptoms, but LLD patients demonstrate residual cognitive impairment. Due to the lack of effective treatments, understanding how risk factors affect the course of AD is essential to manage AD. Advances in neuroimaging, including resting-state functional MRI (fMRI), have been used to address neural systems that contribute to clinical symptoms and functional changes across various psychiatric disorders. Resting-state fMRI studies have contributed to understanding each of the two diseases, but the link between LLD and AD has not been fully elucidated. This review focuses on three crucial and well-established networks in AD and LLD and discusses the impacts on cognitive decline, clinical symptoms, and prognosis. Three networks are the (1) default mode network, (2) executive control network, and (3) salience network. The multiple properties emphasized here, relevant for the hypothesis of the linkage between LLD and AD, will be further developed by ongoing future studies.

## 1. Introduction

Dementia, one of the most common neurodegenerative disorders, is a devastating illness characterized by significant cognitive decline that induces interference in daily life and behavioral disturbances [1]. Alzheimer’s disease (AD) is the most common dementia type, with worldwide patients expected to increase from 82 million in 2030 to 152 million in 2050 [2]. One in every 2–3 people over the age of 85 will develop AD-related dementia [3], and most AD patients experience mild cognitive impairment (MCI), which is the preclinical status of dementia with modest cognitive decline without dysfunction in daily life [4,5]. Several studies have established that the accumulation of amyloid β, hyperphosphorylation of tau proteins, and neuroinflammation affect the neurodegeneration seen in AD [6,7]. However, there is no effective drug for both delaying onset and restoring cognitive function. Therefore, delaying disease onset or progression could provide a significant reduction in the social and economic burden of these diseases [8]. For delaying or preventing AD, previous studies have found several modifiable risk factors, including diet, midlife hypertension, type 2 diabetes mellitus, smoking, cognitive/physical inactivity, traumatic brain injury, and depression [9,10,11].

Depression is the most prevalent coexisting noncognitive feature that occurs along with cognitive deficits and is associated with neurodegenerative disorders and cognitive decline [12,13,14]. Because a major depressive disorder (MDD) is a heterogeneous diagnostic category that features differences in symptom profiles, comorbidities, and the course of disease [15,16], late-life depression (LLD) with an age of depression onset over 60 years has received a great deal of attention [17,18]. Moreover, the global number of individuals with LLD has increased by 27.1% from 2007 to 2017 [19]. Therefore, elucidating the link between the two disorders will help doctors and families understand and manage AD. Epidemiologic data have shown that LLD increases the risk of AD [20,21], and LLD is a risk factor that affects the progression of dementia from the normal cognition to MCI and from MCI to dementia [12,21,22,23]. Additionally, the risk of conversion from MCI to AD may vary due to the symptom severity of LLD or its successful treatment [24]. Individuals with LLD and high amyloid β levels exhibited a shortened conversion time than those without depression and with high amyloid β levels [25,26]. Altered levels and metabolism of amyloid β seen in AD were also reported in individuals with LLD [27]. Although these findings support previously suggested mechanisms that connect depression and dementia [28], a previous systematic review pointed out that these results are not consistent with other studies [29]. This discrepancy may be due to the study population differences or methodologic differences between the various studies [30]. Therefore, it is necessary to subdivide the study population and conduct research associated with more specific criteria.

Cognitive impairments in individuals with depression have been consistently reported in meta-analyses and reviews [31,32,33,34]. Based on these results, difficulties with concentration and making decisions have been described as part of major depressive disorder (MDD) [1]. Cognitive impairments in MDD were reported across most domains [35,36]. These cognitive impairments in MDD patients are usually normalized after remission of the MDD [35,36]. However, studies using a comprehensive neuropsychological battery have reported that cognitive impairment in remitted LLD patients persisted in executive function and episodic memory compared to healthy controls [37,38,39]. In addition, a longitudinal study has reported that LLD patients exhibit a significant decline in all domains, and three-month remitters also exhibited a significant decline in verbal fluency and executive function [17], suggesting that certain aspects of executive functioning are associated with the traits of LLD. Although other studies reported inconsistent results with no difference in LLD [40,41,42], these inconsistent results may be attributed to the differences in cognitive tests. Episodic memory is the other main impaired cognitive domain in individuals with MCI [43]. Impairment in these cognitive domains was usually exhibited to a greater extent in individuals with LLD+MCI (and those with AD), relative to individuals with LLD [44,45,46,47].

In recent years, using improved neuroimaging technology, we can investigate brain structure and function through neuroimaging tools, magnetic resonance imaging (MRI), computed tomography, and positron emission tomography (PET). Among them, functional MRI (fMRI) can provide information about the properties of functional connectivity (FC)—that is, collections of brain regions that are coactivated to support shared functions—during a task or rest (i.e., in the absence of stimuli) through measuring the blood oxygenation level-dependent (BOLD) signal [48,49]. More specifically, previous studies have suggested resting-state (rs)-fMRI as a promising method for investigating the behavioral characteristics including psychological states: sustained attention [50], personality [51], temperament traits [52], creative ability [53], and cognitive ability, such as working memory and motor performance [54]. These newer methods provide reproducible results and reflect stable trait-like neurobiological signatures [55,56]. Recent work also presents that the patterns of resting-state FC are uniquely related both to specific symptoms and to respond to different forms of treatment [57,58]. Thus, reviewing rs-fMRI results seems to be suitable for understanding the links between AD/MCI and LLD.

## 2. Methodological Overview of Resting-State fMRI (rs-fMRI) Studies

Various analytical strategies are available to study resting-state network connectivity [59]. (1) Seed-based analysis is a hypothesis-driven approach when researchers initially select the seed region of interest based on their hypothesis and a calculated brain connectivity map by detecting temporal correlation [48]. Seed-based analyses are attractive for assessing FC changes in small samples with good statistical power; however, whole-brain analyses are required to address a more comprehensive understandings on changes in rs-fMRI [60]. (2) Regional homogeneity (ReHo) evaluates the similarity or synchronization between different time series given a region or given regions and their neighbors [61]. (3) Independent component analysis (ICA) is a more complex approach that decomposes the whole brain into a set of independent components as a functional map [62,63]. (4) Graph theory constructs models of interrelationships (represented by edges) between brain regions (represented by nodes) and assesses the state of the brain network using various measures [64,65]. (5) To address directional interaction within and between functional networks, incorporating resting-state effective connectivity have been conducted [66]. Data-driven techniques such as Granger causal analysis and Bayesian network analysis provide new insights into effective connectivity [66,67]. (6) The amplitude of low-frequency fluctuation (ALFF) and fractional ALFF (fALFF) techniques were developed to assess the spontaneous low frequency (0.01–0.08 Hz) fluctuations in the fMRI signal intensity at rest, which could reflect the intensity of regional brain spontaneous neural activity [68,69].

Several rs-fMRI studies, aiming to unravel the neurobiological mechanisms of depression and dementia, have investigated abnormalities in various structures, including the frontal gyrus, precuneus, cingulate gyrus, parahippocampal cortex, cerebellum, or putamen [70,71,72]. However, recent meta-analyses of these studies did not reveal any significant regional convergence of neuroimaging findings for depression [73,74], suggesting that no single brain region is exclusively responsible for LLD’s heterogeneous symptoms. A behavior or a clinical symptom typically involves synchronizing many brain regions in a network-based fashion [75]. Experiments have identified three major functional networks in LLD, AD, and MCI: (1) the default mode network (DMN), (2) executive control network (ECN), and (3) salience network (SN) [41,76,77,78]. Below, we review rs-fMRI studies in LLD, AD, and MCI patients according to individual neural networks for ease of interpretation of the results associated with cognitive function. The analysis methods for resting-state functional connectivity, reference anatomy used for brain parcellation, types of scanners, and characteristics of groups included in the study are essential pieces of information to understand the study results clearly. Therefore, we presented the table which summarizes sample size, age, study type, scanner type, reference space, and analysis method of each section’s key studies in Supplementary materials.

## 3. The Default Mode Network (DMN)

### 3.1. Overview of DMN

The DMN was initially described as brain regions that consistently showed synchronized deactivation during tasks and activation during rest [79]. This network now generally includes the medial prefrontal cortices (mPFCs), the posterior cingulate cortex (PCC), precuneus, inferior parietal lobule, lateral temporal cortex, and hippocampal formation [80,81]. The DMN is known to be normally deactivated during complex cognitive processing and active during rest, and further studies found that DMN activity is associated with internal processes, such as self-referential thinking [82], autobiographical memory [83], or thinking about the future [84]. Previous meta-analyses, including studies measuring ReHo, ALFF, and fALFF, suggested that altered DMN connectivity seems robust to the choice of analytical methods [85]. The DMN is generally divided into an anterior subdivision centered on the mPFC and a posterior subdivision centered on the PCC and the precuneus cortex [80,86]. Although both the anterior and posterior parts of the DMN are related to spontaneous or self-generated cognition, they seem to be different according to their specific functions [86,87]. Generally, the anterior DMN is more related to self-referential processing and emotion regulation, partly through its strong connections with limbic areas, and the posterior DMN has been implicated in both consciousness and memory processing through its relation to the hippocampal formation [87,88].

### 3.2. rs-fMRI Studies Associated with DMN in Late-Life Depression (LLD)

The fact that DMN is related to processes mostly employed during rest, such as self-generated thought, has gained significant attention, especially with studies related to depression [89]. DMN activity is considered to be negatively correlated to the ECN activity because reducing the brain’s perspective processes seems necessary to focus on the imminent task [84]. In this line, failure to reduce DMN activity has been suggested as a sign of an inability to quiet or inhibit internal mentation or emotional processing [90]. Although not the focus of this review, the relative increases in DMN connectivity during tasks has been consistently reported in various task-based fMRI studies in individuals with depression [91,92]. Several rs-fMRI studies have also reported a relative increase in DMN connectivity [93].

In addition, the difference of connectivity pattern between the anterior and the posterior DMNs in individuals with LLD has been reported. Decreased FC in the posterior DMN have been reported in individuals with LLD compared to healthy controls by rs-fMRI studies using ReHo and ALFF [94,95,96]. Opposing elevation of FC in the anterior DMN was also observed in LLD patients [96]. This difference of FC between the anterior and the posterior DMNs, increased in the anterior DMN and decreased in the posterior DMN, has been also reported in rs-fMRI studies in younger adults with depressed moods compared to healthy controls [77,97]. Although the results in the elderly were not presented, several studies reported that the anterior and the posterior DMNs were associated with different depressive symptoms, rumination, and autobiographical memory, respectively [98]. Moreover, this difference persisted after 12 weeks of antidepressant treatment in young subjects who recovered from MDD [99].

Seed-based analysis that used seed regions of the PCC and precuneus reported interesting results. Unmedicated LLD patients presented with decreased PCC connectivity with increased connectivity in the anterior DMN at baseline. This decrease in connectivity was partly restored after 12 weeks of treatment with paroxetine [100], suggesting that connectivity between the anterior and posterior DMN regions reflects treatment effects. Seed-based analysis presented that the dissociation between the posterior DMN and ECN was also reported in LLD individuals with current depression compared to the healthy control group [92]. During the restoration of this dissociation after antidepressant treatment, it was also reported that the connectivity between PCC and MFG decreased at baseline, then the FC from PCC to the bilateral medial frontal gyrus increased after 12 weeks of antidepressant treatment in LLD patients [101]. In the seed-based analysis using mPFC as a seed region, the dissociation between the anterior and posterior DMNs in depression has been consistently reported in young adults [102,103]. Van Tol et al. (2014) reported increased connectivity between the mPFC and left anterior insula, indicating increased connectivity between the anterior DMN and the SN [103]. We presented key findings associated with LLD in Table 1 and characteristics of main rs-fMRI studies in Supplementary Table S1.

### 3.3. rs-fMRI Studies Associated with DMN in Alzheimer’s Disease (AD) and Mild Cognitive Impairment (MCI)

The DMN has garnered considerable attention in rs-fMRI studies of neurodegenerative diseases, and the findings have been rather consistent. Early rs-fMRI studies focused on the hippocampus [104], because amyloidosis and tau pathology initially appear in the hippocampus [105,106,107], and hippocampal volume loss during the progression of AD is directly associated with cognitive decline in longitudinal studies [108,109]. Various seed-based analyses have reported that less hippocampal FC was found in a broad spectrum of cortical and subcortical regions in AD patients than in healthy individuals [110,111,112]. This altered hippocampal FC has been replicated in more recent seed-based analyses [113,114,115,116,117].

Various rs-fMRI studies that used ICA, fALFF, and ALFF to assess broader networks have also reported consistent results. While there are some inconsistent results in the exact regions reported as being affected by decreased connectivity, there are common significant regions that are nodes of the DMN in AD, including the precuneus and PCC [118,119,120,121,122,123,124,125,126,127,128,129,130,131,132]. Decreased connectivity within the DMN is often accompanied by increased connectivity in the frontoparietal network and SN [133]. In addition to these well-established results of the entire DMN, further studies addressed the dissociation between subdivisions of the DMN (anterior and posterior), suggested by ICA studies in AD patients [120]. There are both results with connectivity reductions mainly in the posterior DMN [134], but with altered connectivity to the anterior DMN [135,136]. In the dissociation between subdivisions of the DMN, an interesting result was reported in longitudinal studies. Findings from patients with early-onset Alzheimer’s disease revealed an increase in the anterior DMN and decreased posterior DMN connectivity [120].

Analysis based on graph theory to assess the alteration of brain networks in AD has shown impressive results. The degree of centrality and clustering coefficients represent the density of a network that is reduced in AD patients [137,138,139,140], and networks in AD had longer distances than healthy controls with the loss of edges [141,142,143]. These studies also reported a negative correlation between small-worldness that reflected a balance between local processing and global integration in the human brain and disease severity [138,139,140]. Similar to overall network changes, small-worldness has been consistently reported in AD patients, asymptomatic apolipoprotein Apo ε4 mutation carriers, and the aging elderly [140,144]. However, inconsistent results have reported an increase in the clustering coefficient in AD compared to healthy subjects [128,143].

Alteration of DMN connectivity is associated with a genetic mutation in AD. In particular, autosomal-dominant mutation carriers (PSEN1, PSEN2, or APP), who were young and asymptomatic, presented with altered DMN connectivity [145,146,147]. Regarding the Apo ε4 allele, various studies have reported diminished DMN connectivity in carriers of at least one Apo ε4 allele in all age ranges [144,146,147,148,149,150,151]. These results suggest some potential for the use of DMN connectivity for early identification of AD in young adults who carry relevant genetic mutations. Moreover, rs-fMRI studies have also reported DMN connectivity changes before the amyloidosis detected by Pittsburgh compound B [152,153,154], which can support the potential of DMN connectivity as an early marker of AD.

The clinical implication of DMN connectivity has been investigated in various areas. Altered DMN connectivity was correlated with the extent of cognitive decline in middle-aged and elderly Apo ε4 allele carriers [155,156,157]. This association has been shown consistently in AD or MCI patients related to global cognition and episodic memory performance [127,158,159,160,161]. With consistent results of altered DMN connectivity in rs-fMRI studies, attention has been focused on how these alterations can be counteracted by treatment [78]. Studies on donepezil’s effect on the resting-state networks in AD have found that the application of donepezil leads to an increase in previously reduced connectivity with no differences in study groups at baseline [162,163].

Additionally, altered connectivity between the anterior and posterior DMNs is associated with aging and age-related cognitive decline [147,164]. This dissociation in DMN subdivision has also been shown in the cognitively normal elderly who presented with abnormal cerebrospinal fluid amyloid or tau proteins [165], or cerebral amyloidosis detected by PET [166]. These results are congruent with the idea that AD patients have a long preclinical period with functional alterations before the onset of disease symptoms. For the network connectivity changes in the progression of AD, longitudinal studies reported decreased connectivity between the precuneus and ECN [167], different local aging patterns in the FC between the left hippocampus and the PCC [168], and decreased global connectivity associated with the striatum [169]. Based on the suggested potential of DMN to provide biomarkers, several rs-fMRI studies have addressed early detection, classification, and prediction in AD and MCI. These studies have shown relatively high performances: ICA [161,170,171,172,173], seed-based analysis [174], and graph theory [175]. We presented key findings associated with AD and MCI in Table 2 and characteristics of main rs-fMRI studies in Supplementary Tables S2 and S3 .

## 4. The Executive Control Network (ECN)

### 4.1. Overview of ECN

The ECN, a functionally linked system, consists of brain structure cores that include the dorsolateral prefrontal cortex (dlPFC), medial frontal cortex, lateral parietal cortex, cerebellum, and supplementary motor area [176]. Initially, studies investigating executive function using task-based fMRI identified the coactivation patterns of an ECN during executive function tasks [177]. Beyond task-based fMRI, rs-fMRI studies, and structural MRI studies have also identified an ECN [176,178]. Moreover, a close correlation between executive function changes with aging and alterations in the ECN have been reported [179]. This correlation has been reported in studies that used the ECN to study the functional mechanisms of executive function changes in patients with psychiatric disorders, Parkinson’s disease [180], MCI [181], AD [182], and LLD [183]. 

### 4.2. rs-fMRI Studies Associated with ECN in LLD

Disruption of the ECN in LLD patients with current depression symptoms has been consistently reported compared to healthy controls [184,185]. Particularly, seed-based analyses using the dlPFC as the seed region demonstrated decreased FC in the frontoparietal areas in LLD individuals with current depression [41]. Other studies using the cerebellum as a seed region reported decreased FC in ECN nodes, including in dlPFC and the parietal cortex, as well as DMN nodes [186,187]. Studies using ICA analysis presented different connectivity patterns for each region in the ECN, with increased FC in the inferior parietal but decreased FC in the dlPFC and superior frontal areas [39]. This decreased connectivity associated with the ECN has been consistently presented in other rs-fMRI studies using ReHo [94,188] and ALFF [40]. Additionally, LLD remitters also demonstrated decreased FC in the frontal-parietal cortex 3 months after remission [189]. After 21 months, individuals with remitted LLD presented a return to decreased FC. 

Executive dysfunction is a common symptom in LLD patients. About 30 to 40% of nondemented elders with LLD demonstrate executive dysfunction during neuropsychological tests [190]. Disruption of the ECN was associated with executive dysfunction that included susceptibility to distraction, an inability to sustain attention, poor multitasking, organizational difficulties, and concrete or rigid thinking [191]. A recent study reported that LLD patients’ FC between the dlPFC and other bilateral regions was negatively associated with executive function in LLD subjects [192]. Researchers reported that executive dysfunction is associated with greater functional disability levels in LLD [193,194]. Deficits in word-list generation and response inhibition that represent executive function predict poor and slow antidepressant responses and relapses [195,196]. In this regard, the ECN seems to be related to the LLD’s clinical prognosis associated with executive dysfunction. We presented key findings associated with LLD in Table 3 and characteristics of main rs-fMRI studies in Supplementary Table S4.

### 4.3. rs-fMRI studies associated with the ECN in AD and MCI

Additionally, in AD and MCI, rs-fMRI studies using the ICA analysis identified a significant difference in ECN connectivity across AD and MCI patients and normal controls [197]. In the case of intraconnectivity of the ECN, results seem inconclusive, with some studies reporting no changes in AD patients and others reporting increased connectivity [121,127,198]. However, studies using seed-based analysis consistently reported abnormal FCs between the hippocampus and nodes of the ECN. Previous studies have demonstrated that functional brain activity within portions of the ECN was abnormal in patients with MCI and AD [182,199]. Specifically, the directed FCs from the left hippocampus to the right superior frontal gyrus (SFG) and left medial frontal gyrus (MFG) to the right hippocampus were significantly decreased in MCI or AD patients. The SFG [175] and the MFG [200,201] are essential components of the dlPFC that play essential roles in the ECN.

Moreover, Cai et al. (2017) reported different effective connectivity patterns for the ECN in normal controls and three subgroups of MCI: (1) MCI-R—MCI reverted to the normal functioning state and stabilized to the normal state in 24 months; (2) MCI-S—MCI patients who remained in a stable disease state for 24 months; (3) MCI-P—MCI that progressed to AD and stabilized to AD in 24 months. In this study, the effective connectivity patterns in the ECN were less disrupted and less obvious among MCI-R and MCI-S to MCI-P. In addition, ECN connectivity strengths were not changed in MCI-R patients and normal controls compared to MCI-S and MCI-P patients [181]. These results suggest the importance of the ECN in dementia progression from MCI to AD. We presented key findings associated with AD and MCI in Table 4 and characteristics of main rs-fMRI studies in Supplementary Table S5.

## 5. The Salience Network (SN)

### 5.1. Overview of SN

The SN is the brain network that detects and filters external stimuli and recruits relevant functional networks [202]. This network is essential for detecting and integrating emotional and sensory stimuli, allocating attention, and switching between internally directed cognition and externally directed cognition [203]. The SN’s hub is the ventral anterior insula [204], and the SN also includes nodes in the amygdala, hypothalamus, ventral striatum, and thalamus [203]. The SN was suggested to be functionally subdivided into dorsal and ventral components that support cognitive and emotional controls, respectively [205]. The key SN regions activated during cognitive tasks consist of dorsal components: the dorsal anterior cingulate cortex and the right anterior insula [205,206]. For example, the SN engages the ECN and disengages the DMN during cognitive tasks but does the opposite during rest [207]. Regarding cognitive function, the extent of dissociation between the ECN and SN is related to cognitive task performance [208]. Additionally, the structural connectivity shown by diffusion tensor image analysis is also positively correlated with SN intraconnectivity (right anterior insula to dorsal anterior cingulate cortex) and deactivation of the DMN during tasks, which is in turn related to cognitive function [209].

### 5.2. rs-fMRI Studies Associated with the SN in LLD

A disrupted standard pattern of SN connectivity is suggested to be one of the key traits of the pathogenesis of depression, particularly in the insula and amygdala [210]. Elevated connectivity between the insula and DMN was enhanced in MDD patients, which may hinder the above standard pattern [91]. The FC from the amygdala, another important SN node, to the hippocampus was decreased in adolescents with depression and at a high risk of depression [211,212]. Additionally, seed-based analysis in younger adults using the amygdala as a seed region was positively associated with increased amygdala FC with DMN nodes and long-term negative emotions [213]. One study that addressed apathy in LLD patients found that LLD patients with apathy exhibit increased FC between the SN and DMN compared with nonapathetic elders with depression [77]. Overall, these results may suggest that increased FC between the SN and DMN may predispose individuals to depression and is further correlated with vegetative symptoms in LLD [186]. However, inconsistent results for decreased FC between the amygdala and precuneus in depressed patients compared with controls have been reported [214]. 

Network analysis reported that elders with LLD also demonstrate a decreased negative FC between the SN and ECN compared to nondepressed age-matched controls [39]. Another study that compared correlation patterns among significant brain networks in LLD patients compared to nondepressed elderly controls reported dissociation patterns among the ECN/SN, and DMN observed in controls [215]. These results represent a failure of internetwork cohesiveness in LLD [185]. Moreover, decreased negative FC between the ECN and the SN was associated with cognitive impairment and severity of depression symptoms in LLD patients [39]. In addition, a worse treatment response to antidepressants was also associated with this disrupted standard SN pattern [216]. We presented key findings associated with LLD in Table 5 and characteristics of main rs-fMRI studies in Supplementary Table S6.

### 5.3. rs-fMRI Studies Associated with SN in AD and MCI

SN connectivity has increasingly gained attention from researchers who address neurodegenerative disease [133]. Although intensified SN connectivity was observed in AD patients compared to healthy controls in ICA studies [130,170], another ICA study in AD patients found contradictory evidence of a decrease in dorsal SN [121]. This increased SN connectivity has been consistently reported in cognitively normal individuals with elevated amyloid levels [166,217], Apo ε4 carriers [156,218], and MCI patients [74]. Moreover, studies that have addressed both amyloid and tau within the DMN and SN reported interesting results, with increased connectivity in the SN and DMN in individuals with elevated amyloid but little evidence of tau, but decreased connectivity in the SN and DMN in individuals with both elevated tau and amyloid levels [219]. These findings highlight the point that SN connectivity changes occur in preclinical dementia, and SN connectivity may change with disease progression. We presented key findings associated with AD and MCI in Table 6 and characteristics of main rs-fMRI studies in Supplementary Table S7.

## 6. Conclusions

**Figure 1:**
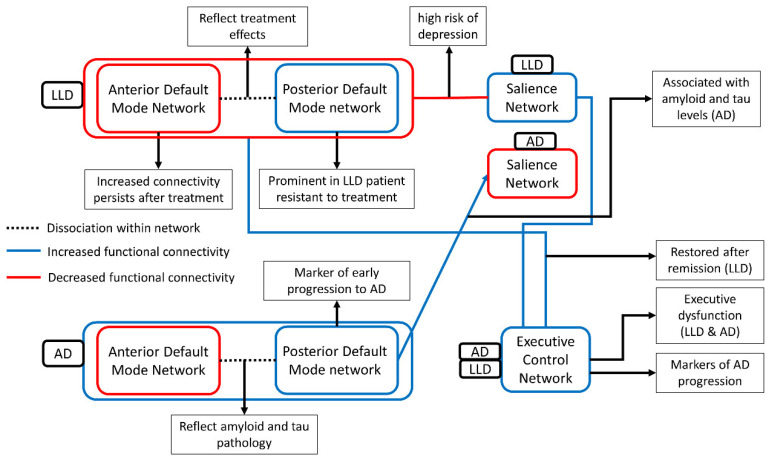
The figure presents the aberrant functional connectivity between three networks in AD and LLD and its clinical implication. Blue line and red line indicate the decreased functional connectivity and the increased functional connectivity compared with healthy control, respectively. Decreased functional connectivity of the executive control network was commonly observed in AD and LLD [192,197]. In contrast, the functional connectivity of the salience network and the default mode network were differently altered. The default mode network connectivity decreased in AD and increased in LLD [93,127], and the salience network increased in AD and decreased in LLD [130,215]. However, dissociated functional connectivity pattern in DMN, increased in the anterior DMN and decreased in the posterior DMN, was commonly observed in both AD and LLD [96,166]. This dissociation reflects treatment effects in LLD and amyloid/tau pathology in AD [100,165]. This similarity of dissociation seems to be a possible mechanism of association between LLD and AD highlighted in epidemiological studies.

Alteration in brain networks during the resting state contributes to the symptoms and progression of LLD and AD. Above, we described LLD and AD, focusing on key networks known to be necessary for the network-level description of these two diseases: the DMN, ECN, and SN (Figure 1). A growing body of literature suggests an opposite direction for overall DMN alterations in LLD and AD, with increased connectivity of the DMN in LLD but decreased DMN connectivity in AD. However, the dissociation between the anterior DMN and posterior DMN provides insight into the link between depression and dementia. In the early stage of AD, the alteration in the DMN is different between its anterior and posterior subdivisions, with increased anterior DMN connectivity, and decreased posterior DMN connectivity [120]. Similar dissociation patterns were also observed in individuals with depression, and this increased anterior DMN persists after antidepressant treatments [99]. Additionally, a posterior DMN connectivity reduction was observed in individuals with LLD + MCI compared to LLD only [46,188] and also in LLD patients with an inadequate response to treatment [101]. Additionally, the PCC, the hub of the posterior DMN, is a marker of very early AD progression, as consistently seen with T1-weighted imaging, postmortems, and PET studies [220,221,222]. Although this association between the dissociation of DMN connectivity and AD and LLD remains to be explored, severe depression may induce the clinical manifestation of cognitive impairment or the onset of eventual cognitive decline, a signal of intrinsic network dysfunction.

Regarding the ECN, both AD and LLD exhibit disrupted ECN connectivity. As discussed, executive dysfunction associated with disrupted ECN connectivity seems to be related to the clinical prognosis of LLD with poor and slow antidepressant responses and a high relapse rate. The findings that the degree of ECN disruption is associated with cognitive decline 24 months after MCI is also covered above. With the hypothesis that depression precedes cognitive decline or induces cognitive decline [223], these results suggest the possibility that the ECN is a target that can modify the impact of LLD on cognitive declines. A noninvasive treatment is being conducted with the ECN as a target [224].

Another interesting issue seen in rs-fMRI studies is associated with the pathogenic process. Several studies using rs-fMRI associated with AD, tau, and amyloid pathology consistently reported that the spreading of these pathologies throughout the brain correlates to brain network disruption, as discussed in this review. Because the DMN, ECN, and SN are multimodal networks that are metabolically expensive and display high rates of cerebral blood flow, aerobic glycolysis, and oxidative glucose metabolism [225], these networks may be vulnerable to AD-associated pathogenic processes. Although spatial deposition patterns have been not convergent, there has been a recent observation that tau and amyloid plaques overlap with brain tissue loss in hub regions of these discussed brain networks [226]. Another review also points to this correlation and suggests that AD-associated pathophysiological processes may explain changes in these networks [133].

Despite the consistent findings across studies, several critical knowledge gaps remain. The lack of standardized protocol for addressing the brain using rs-fMRI has not been adequately addressed. Regarding preprocessing steps of fMRI for dealing with noise, preprocessing steps for rs-fMRI data have evolved to be more diverse than preprocessing for task-based fMRI data. With the diversity of statistical approaches applied to the purified data, these nonstandardized various methods make comparisons across studies extremely difficult. Even if the same terms are used to describe results such as network strength or connectivity, one method’s results cannot be compared well with the results of studies using other technologies.

Additionally, our literature did not include task-based fMRI studies in AD and LDD, which clearly expressed the need for additional research. Compared to rs-fMRI studies, a task-based fMRI study is a relatively conventional approach and is challenging to perform due to the needs of involving tasks. Nevertheless, preprocessing steps and statistical methods for task-based fMRI have been more standardized than those for rs-fMRI and have less influenced results, where an external behavioral standard is available. Because task-based fMRI studies are more clinically interpretable, future studies that include tailored tasks concerning specific cognitive, emotional, and social functions would expand our knowledge of AD and LLD.

Recent studies using directed graph theory or combining multiple imaging tools have presented promising results in the field of diagnosis and prediction [227,228]. Therefore, future studies combining multimodal imaging tools such as PET, MRI, and fMRI in AD and LLD patients samples with special considerations such as age, sex, age of onset, treatment outcomes, the severity of illness, and cognitive impairment would help us understand the fundamental functional pathological changes in AD and LLD. Longitudinal studies that include various treatment tools would also help uncover the association between depression and AD-associated pathophysiological processes. Standardized protocols in fMRI data collection and analysis would be helpful to reduce heterogeneity across these physiological states.

## Figures and Tables

**Table 1:** Summary of key findings of resting-state functional MRI (rs-fMRI) studies associated with the default mode network (DMN) in late-life depression (LLD) patients included in the review.

Summary of Key Findings	Key Studies
Relative increase in DMN functional connectivity	[92]
Dissociation within DMN network - decreased posterior DMN functional connectivity - elevation anterior DMN functional connectivity	[94,95,96]
Restoration of dissociation within DMN network was associated with antidepressant treatment	[100,101]

Abbreviations: DMN, Default mode network.

**Table 2:** Summary of key findings of rs-fMRI studies associated with the default mode network (DMN) in Alzheimer’s disease (AD) and mild cognitive impairment (MCI) patients included in the review.

Summary of Key Findings	Key Studies
Decreased in DMN functional connectivity	[112,118,123,126,127,128,131]
Dissociation within DMN network; - decreased posterior DMN functional connectivity - elevation anterior DMN functional connectivity	[134,135]
DMN networks had longer distances with the loss of edges	[138,141,142]
Altered DMN functional connectivity was associated with decline of cognition	[143,158,160]
Altered DMN functional connectivity was associated with genetic mutation	[146,149,152,154,157,163]

Abbreviations: DMN, default mode network.

**Table 3:** Summary of key findings of rs-fMRI studies associated with the executive control network (ECN) in late-life depression (LLD) patients included in the review.

Summary of Key Findings	Key Studies
Decreased in ECN functional connectivity	[186,188]
Restoration of ECN functional connectivity after remission	[189]
Decreased in ECN functional connectivity was associated with executive dysfunction	[192]

Abbreviations: ECN, executive control network.

**Table 4:** Summary of key findings of rs-fMRI studies associated with the executive control network (ECN) in Alzheimer’s disease (AD) and mild cognitive impairment (MCI) patients included in the review.

Summary of Key Findings	Key Studies
Decreased in ECN functional connectivity	[197]
Inconclusive result was also reported (increased ECN functional connectivity in AD)	[198]
ECN functional connectivity was associated with AD progression	[181]

Abbreviations: ECN, executive control network; AD, Alzheimer’s disease.

**Table 5:** Summary of key findings of rs-fMRI studies associated with the salience network (SN) in late-life depression (LLD) patients included in the review.

Summary of Key Findings	Key Studies
Decreased SN functional connectivity	[39]
Increased functional connectivity between SN and DMN	[77,215]
Disrupted SN pattern was associated with worse treatment response	[216]

Abbreviations: DMN, default mode network; SN, salience network.

**Table 6:** Summary of key findings of main rs-fMRI studies associated with the salience network (SN) in Alzheimer’s disease (AD) and mild cognitive impairment (MCI) patients included in the review.

Summary of Key Findings	Key Studies
Intensified SN functional connectivity was observed in AD patients	[170]
Increased SN functional connectivity was associated with - elevation of amyloid level, Apo ε4 carriers, and elevation of tau	[166,217,218, 219]

Abbreviations: SN, salience network; AD, Alzheimer’s disease.

## Data Availability

No new data were created or analyzed in this study. Data sharing is not applicable to this article.

## Supplementary Materials

The following are available online at https://www.mdpi.com/2227-9059/9/1/82/s1.
