# Supplementary material for: Crosstalk between Depression and Dementia with Resting-State fMRI Studies and Its Relationship with Cognitive Functioning
Source: Biomedicines. 2021 Jan 16;9(1):82. doi: 10.3390/biomedicines9010082 (PMC7830949; doi:10.3390/biomedicines9010082)
Supplement: Supplementary file 1 [file biomedicines-09-00082-s001.pdf]

# Crosstalk between Depression and Dementia with Resting-State fMRI Studies and Its Relationship with Cognitive Functioning

**Junhyung Kim and Yong-Ku Kim**

## (Supplementary Information)

**Supplementary Table S1.** Characteristics of main rs-fMRI studies associated with the default mode network (DMN) in late-life depression (LLD) patients included in the review.

| References | Experimental Details | | | | | | |
|---|---|---|---|---|---|---|---|
| | LLD | | Healthy Control | | | | |
| | Sample Size (Male) | Age Mean (SD) | Sample Size (Male) | Study Type | Scanner | Reference Space | Analysis Method |
| [92] | 32(11) | 67.2(5.8) | 39(19) | CS | 3.0T S | MNI | SB |
| [94] | 18(8) | 67.2(7.3) | 14(7) | CS | 1.5T GE | TAL | ReHo |
| [95] | 15(6) | 67.5(6.1) | 15(6) | CS | 1.5T GE | MNI | ReHo |
| [96] | 52(14) | 71.4(7.6) | 36(9) | CS | 3.0T S | MNI | ALFF |
| [100] | 12(5) | 70.5(4.9) | 8(3) | CS | 1.5T GE | MNI | SB |
| [101] | 47(13) | 68.7(6.9) | 46(13) | Cohort | 3.0T S | Colin27 | SB |

Abbreviations: ALFF, Amplitude of low-frequency fluctuation; CS, cross-sectional study; GE, General Electronics; MNI, Montreal Neurological Institute; ReHo, Regional homogeneity; S, Siemens; SB, seed-based analysis; SD, standard deviation; T, tesla; TAL, Talairach.

**Supplementary Table S2**. Characteristics of main rs-fMRI studies associated with the default mode network (DMN) in Alzheimer's disease (AD) and mild cognitive impairment (MCI) patients included in the review.

| References | Experimental Details | | | | | | | | |
|---|---|---|---|---|---|---|---|---|---|
| | AD | | MCI | | Healthy Control | | | | |
| | Sample Size (Male) | Age Mean (SD) | Sample Size (Male) | Age Mean (SD) | Sample Size (Male) | Study Type | Scanner | Reference Space | Analysis Method |
| [112] | 13(5) | 70.1(6.7) | N/A | N/A | 8(3) | CS | 1.5T S | TAL | SB |
| [118] | 13(8) | 74.5(9.7) | 12(6) | 69.1(7.4) | 13(5) | CS | 1.5T S | MNI | ICA |
| [123] | 23(7) | 73(9) | 19(10) | 76(8) | 21(12) | CS | 3.0T GE | MNI | ReHo |
| [126] | 39(23) | 67(8) | 23(15) | 71(8) | 43(22) | CS | 1.5T S | MNI | ICA |
| [128] | 21(9) | 64.2(8.7) | N/A | N/A | 18(12) | Cohort | 3.0T GE | MNI | ICA |
| [131] | 11(7) | 71.9(7.9) | 10(6) | 71.2(4.1) | 10(7) | CS | 3.0T S | MNI | ICA |
| [134] | 24(14) | 68.2(8.4) | N/A | N/A | 16(7) | CS | 3.0T P | MNI | ICA |
| [135] | 28(18) | 78(4) | N/A | N/A | 56(36) | CS | 3.0T GE | MNI | ICA/SB |
| [141] | 18(9) | 70.7(7.2) | N/A | N/A | 21(8) | CS | 1.5T S | MNI | Graph |
| [142] | N/A | N/A | 37(17) | 66.8(9.4) | 47(20) | CS | 3.0T S | MNI | Graph |
| [143] | 33(13) | 66.2(9.6) | N/A | N/A | 20(10) | CS | 1.5T M | MNI | Graph |
| [158] | 16(6) | 71.6(5.1) | N/A | N/A | 16(7) | CS | 1.5T P | MNI | SB |
| [160] | N/A | N/A | 30(15) | 72.5(4.4) | 26(12) | CS | 1.5T GE | MNI | SB |

Abbreviations: AD, Alzheimer's disease; CS, cross-sectional study; GE, General Electronics; Graph, Graph analysis; ICA, Independent Component Analysis; M, Marconi; MCI, Mild cognitive impairment; MNI, Montreal Neurological Institute; P, Philips; ReHo, Regional homogeneity; S, Siemens; SB, seed-based analysis; SD, standard deviation; T, tesla; TAL, Talairach.

**Supplementary Table S3.** Characteristics of main rs-fMRI studies associated with the default mode network (DMN) in Alzheimer's disease (AD) and mild cognitive impairment (MCI) patients included in the review (Continued).

| References | Demographic Group: Sample Size, N (Male); Age, Mean (SD) | Study Type | Scanner | Reference Space | Analysis Method |
|---|---|---|---|---|---|
| [127] | Sample size: 570(268)<br>CDR 0: 386; CDR 0.5: 91; CDR 1: 33 | CS | 3.0T S | MNI | SB |
| [138] | CDR 1: Sample size, 31(13); age, 70.7(11.4)<br>CDR 0.5: Sample size, 90(36); age, 74.5(7.5)<br>CDR 0: Sample size, 205(66); age, 66.4(9.8) | CS | 3.0T S | MNI | Graph |
| [146] | CDR 0M-: Sample size, 37(17); age, 38.9(9.7)<br>CDR 0M+: Sample size, 44(17); age, 34.6(8.0)<br>CDR 0.5M+: Sample size, 24(8); age, 44.5(11.7)<br>AD, CDR 1-2M+: Sample size, 15(6); age, 49.3(9.7) | CS | 3.0T S | MNI | ICA |
| [149] | ADAD<br>CDR 0M-: Sample size, 25(10); age, 30.9(10)<br>CDR 0M+: Sample size, 31(12); age, 33.9(8.5)<br>CDR 0 M+: Sample size, 15(5); age, 41.4(10.4)<br>CDR 0.5 M+: Sample size, 8(5); age, 49.4(8.7)<br>LOAD<br>CDR 0: Sample size, 343(117); age, 68.7(9.5)<br>CDR 0.5: Sample size, 74(38); age, 74.0(7.7)<br>CDR 1: Sample size, 27 10); age, 70.1(11.4) | CS | 3.0T S | MNI | SB |
| [152] | Apo ε4 -: Sample size, 62(19); age, 63.3(7.4)<br>Apo ε4 +: Sample size, 38(9); age, 58.8(8.5) | CS | 3.0T S | TAL | SB |
| [154] | Apo ε4 -: Sample size, 18(10); age, 28.6(3.9)<br>Apo ε4 +: Sample size, 18(7); age, 28.4(4.9) | CS | 3.0T S | HOB | ICA |
| [157] | Apo ε4 -: Sample size, 62(21); age, 64.4(7.0)<br>Apo ε4 +: Sample size, 33(13); age, 62.6(7.6) | CS | 1.5T GE | MNI | ICA |
| [163] | mild AD: Sample size, 12(8); age, 77.6(7.1) compare baseline and after treatment | Cohort | 3.0T GE | TAL | SB |

Abbreviations: AD, Alzheimer's disease; ADAD, autosomal dominant Alzheimer's disease; Apo, apolipoprotein; CDR, Clinical Dementia Rating Scale; CS, cross-sectional study; GE, General Electronics; Graph, Graph analysis; HOB, Harvard-Oxford brain; ICA, Independent Component Analysis; LOAD, late-onset Alzheimer's disease; M-, mutation negative; M+, mutation positive; MCI, Mild cognitive impairment; MNI, Montreal Neurological Institute; S, Siemens; SB, seed-based analysis; SD, standard deviation; T, tesla; TAL, Talairach.

**Supplementary Table S4.** Characteristics of main rs-fMRI studies associated with the executive control network (ECN) in late-life depression (LLD) patients included in the review.

| References | Experimental Details | | | | | | |
|---|---|---|---|---|---|---|---|
| | LLD | | Healthy Control | | | | |
| | Sample Size (Male) | Age Mean (SD) | Sample Size (Male) | Study Type | Scanner | Reference Space | Analysis Method |
| [186] | 11(1) | 64.9(4.5) | 18(7) | CS | 3.0T GE | MNI | SB |
| [188] | 22(7) | 67.6(5.5) | 22(9) | CS | 3.0T GE | MNI | ReHo |
| [189] | 14(7) | 67.6(4.0) | 18(1) | Cohort | 1.5T GE | MNI | SB |
| [192] | 79(52) | 66.3(5.9) | 21(9) | CS | 3.0T P | MNI | SB |

Abbreviations: CS, cross-sectional study; GE, General Electronics; MNI, Montreal Neurological Institute; ReHo, Regional homogeneity; SB, seed-based analysis; SD, standard deviation; T, tesla.

**Supplementary Table S5.** Characteristics of main rs-fMRI studies associated with the executive control network (ECN) in Alzheimer's disease (AD) and mild cognitive impairment (MCI) patients included in the review.

| References | Demographic Group: Sample Size, N (Male); Age, Mean (SD) | Study Type | Scanner | Reference Space | Analysis Method |
|---|---|---|---|---|---|
| [181] | MCI-R: Sample size, 13(6); age, 69.8(6.0);<br>MCI-S: Sample size, 33(17); age, 71.0(6.4)<br>MCI-P: Sample size, 19(9); age, 71.1(7.2)<br>HC: Sample size, 36(18); age, 70.8(1.1) | Cohort | 3.0T P | MNI | ICA |
| [197] | AD: Sample size, 14(5); age, 70.4(5.6)<br>MCI: Sample size, 12(4); age, 73.6(6.2)<br>HC: Sample size, 16(4); age, 69(5.1) | CS | 1.5T P | MNI | ICA |
| [198] | mild AD/MCI: Sample size, 13(9); age, 72(9)<br>HC: Sample size, 12(7); age, 72(7) | CS | 1.5T S | MNI | ICA |

Abbreviations: AD, Alzheimer's disease; CS, cross-sectional study; ICA, Independent Component Analysis; MCI, Mild cognitive impairment; MCI-R, MCI patients reversed to normal states and stabilized to a normal condition for 24 months; MCI-S, MCI patients stabilized in the MCI condition for 24 months; MCI-P, MCI patients progressed to AD and stabilized in the AD condition for 24 months; MNI, Montreal Neurological Institute; P, Philips; S, Siemens; T, tesla.

**Supplementary Table S6.** Characteristics of main rs-fMRI studies associated with the salience network (SN) in late-life depression (LLD) patients included in the review.

| References | Experimental Details | | | | | | |
|---|---|---|---|---|---|---|---|
| | LLD | | Healthy Control | | | | |
| | Sample Size (male) | Age Mean (SD) | Sample Size (Male) | Study Type | Scanner | Reference Space | Analysis Method |
| [39] | 39(9) | 68.6(6.5) | 29(12) | CS | 3.0T GE | MNI | ICA |
| [77] | A-LLD: Sample size, 7, age, 69.9(4.9)<br>NA-LLD: 9, 68.3(6.1)<br>HC: 10, 68.6(7.0) | | | CS | 1.5T S | MNI | SB |
| [215] | 32(14) | 68.3(6.5) | 32(13) | CS | 3.0T GE | MNI | ICA |
| [216] | LLD: Sample size, 71(48); age, 71.0(6.6) compare baseline and after treatment | | | Cohort | 3.0T S | MNI | SB |

Abbreviations: A-LLD, apathetic LLD; ICA, Independent Component Analysis; CS, cross-sectional study; GE, General Electronics; MNI, Montreal Neurological Institute; NA-LLD, non-apathetic LLD; SB, seed-based analysis; SD, standard deviation; T, tesla.

**Supplementary Table S7.** Characteristics of main rs-fMRI studies associated with the salience network (SN) in Alzheimer's disease (AD) and mild cognitive impairment (MCI) patients included in the review.

| References | Demographic Group: Sample Size, N (Male); Age, Mean (SD) | Study Type | Scanner | Reference Space | Analysis Method |
|---|---|---|---|---|---|
| [166] | Old: Sample size, 20(6); age, 73.9(8.2)<br>Young: Sample size, 20(6); age, 73.9(8.2) | Regression analysis | 1.5T S | MNI | ICA |
| [170] | AD: Sample size, 12(5); age, 63.3(7.7)<br>bvFTD: Sample size, 12(6); age, 60.8(4.6)<br>HC: Sample size, 12(5); age, 62.0(8.2); | CS | 1.5T S | MNI | ICA |
| [217] | PiB-: Sample size, 26(14); age, 81.2(5.9)<br>PiB+: Sample size, 13(7); age, 83.8(8.0) | CS | 3.0T P | MNI | SB |
| [218] | CN, Apo ε4 --: Sample size, 56(35); age, 79(N/A)<br>CN, Apo ε4+-: Sample size, 26(14); age, 78(N/A) | CS | N/A | MNI | ICA/SB |
| [219] | AD: Sample size, 35(17); age, 65.8(8.3)<br>aMCI: Sample size, 18(10); age, 70.2(7.9)<br>HC, Y: Sample size, 75(38); age, 23.8(4.0)<br>HC, O: Sample size, 21(7); age, 65.0(8.2) | CS | 3.0T S | MNI | ICA |

a. Regression analysis within- and between-network FC are related to both global and regional Aβ deposition. Abbreviations: AD, Alzheimer's disease; Apo, apolipoprotein; bvFTD, behavior variant Frontal Temporal Dementia; CN, cognitively normal; CS, cross-sectional study; ICA, Independent Component Analysis; aMCI, amnestic Mild cognitive impairment; MNI, Montreal Neurological Institute; O, old; P, Philips; PiB-, regional beta-amyloid load (the distribution volume ration, DVR) <1.061; PiB+, regional beta-amyloid load (the distribution volume ration, DVR) >1.061; S, Siemens; T, tesla; Y, young.
